# Case report: ‘Photodynamics of Subependymal Giant Cell Astrocytoma with 5-Aminolevulinic acid’

**DOI:** 10.3389/fsurg.2022.1065979

**Published:** 2023-01-06

**Authors:** Imran Ghani, Sabina Patel, Prajwal Ghimire, Istvan Bodi, Ranjeev Bhangoo, Francesco Vergani, Keyoumars Ashkan, Jose Pedro Lavrador

**Affiliations:** ^1^ King's Neuro Lab, Department of Neurosurgery, London, United Kingdom; ^2^ School of Biomedical Engineering and Imaging Studies, King's College London, London, United Kingdom; ^3^ Department of Neuropathology, King's College Hospital NHS Foundation Trust, London, United Kingdom; ^4^ Department of Neurosurgery, King's College Hospital NHS Foundation Trust, London, United Kingdom

**Keywords:** 5-aminolevulinic acid, fluorescence, tuberous sclerosis, subependymal giant cell astrocytoma, low grade glioma, minimal invasive parafascicular approach

## Abstract

Subependymal Giant Cell Astrocytoma (SEGA) is a common diagnosis in patients with Tuberous Sclerosis. Although surgical treatment is often required, resection may entail a significant risk for cognitive function given the anatomical relation with critical structures such as the fornices and subgenual area. Therefore, target subtotal resections using minimal invasive approaches focused in the higher metabolic areas are valuable options to preserve quality of life while addressing specific problems caused by the tumor, such as hydrocephalus or progressive growth of a specific component of the tumor. In this report, the authors explore the potential role of 5-ALA in the identification of highly metabolic areas during SEGA resection in the context of minimal invasive approaches.

## Introduction

Subependymal Giant Cell Astrocytoma (SEGA) is a low-grade tumor (WHO Grade I) (1) predominantly associated with tuberous sclerosis (2–4). It characteristically arises from the wall of lateral ventricles, and while they are often asymptomatic, symptoms and neurological deterioration can occur as a result of obstructive hydrocephalus.

On imaging, SEGA classically appears as a paraventricular mass near the foramen of Monro, larger than 1 cm, showing calcifications, heterogeneous MRI signal, and marked contrast enhancement. Surgical resection is usually curative for such tumors except when there are multiple tumors in which case medical treatment is favored (5–8).

The value of 5-aminolevulinic acid (5-ALA) for resection of high grade or malignant astrocytomas has been established in recent years (9, 10). However, its use remains controversial in the context of low-grade gliomas, with limited data available (11). While the actual mechanism for 5-ALA uptake by the cells is not completely understood, disturbances in the heme group synthesis pathway, disturbances in the blood-brain barrier, and increase aquaporin expression have all been implicated, although there remains a lack of evidence for a single or driving mechanism (12).

In this report, the authors report the usefulness of 5-ALA assisted resection in a SEGA and perform a review of the existing literature.

## Case description

**Figure 1:**
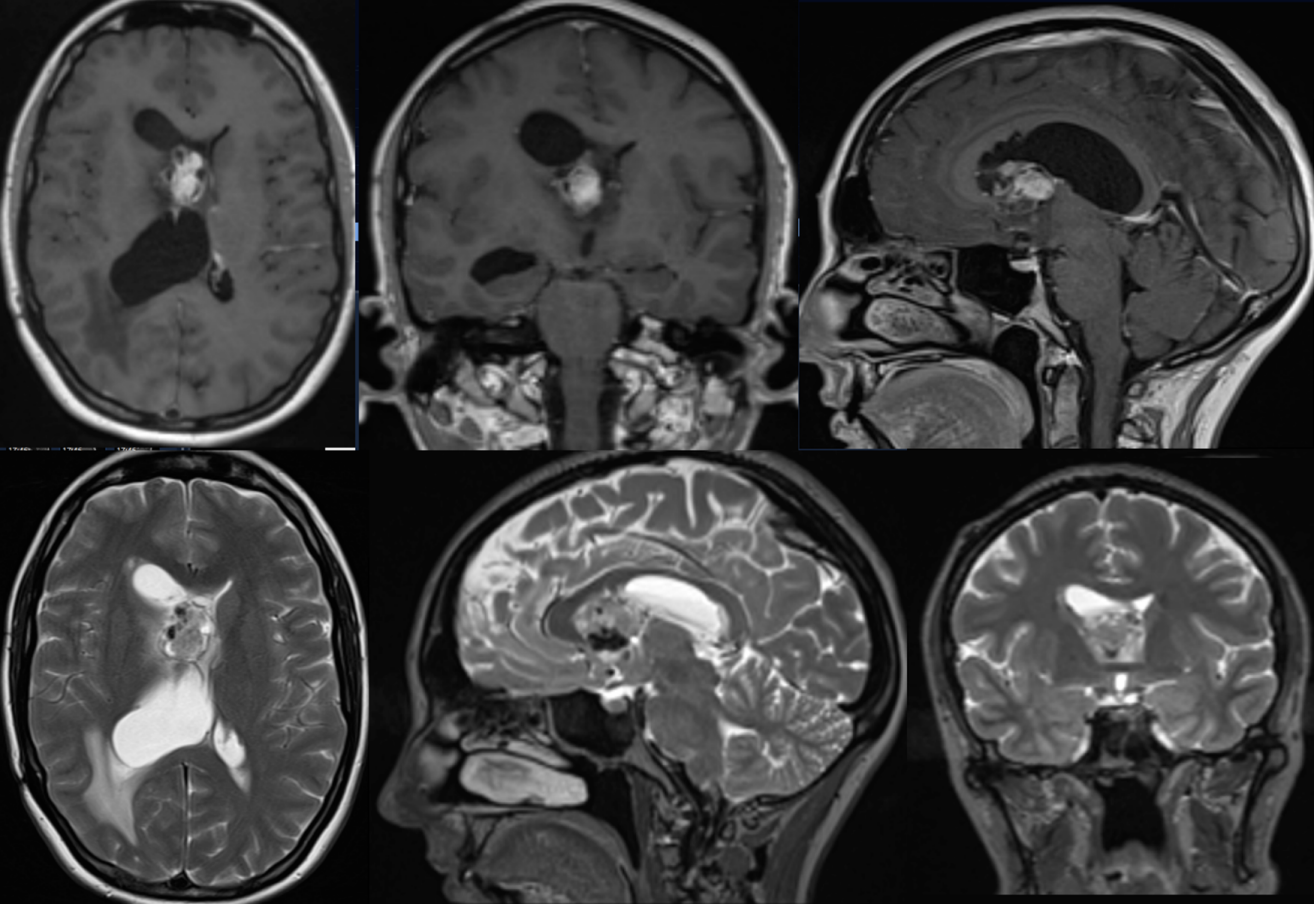
Pre-op MRI scan with T1 post gad and T2 weighted images showing right sided heterogeneous intraventricular lesion with low T2 signal likely calcified components with some areas of avid gadolinium enhancement.

A 32 years old lady, with a background of tuberous sclerosis, was referred to the neuro-oncology service with an incidental finding of SEGA during investigation for migraine (Figure 1). She presented with worsening headaches secondary to obstructive hydrocephalus, which was successfully treated with the insertion of a Ventriculo-Peritoneal shunt (VPS). She remained under surveillance with regular imaging for the underlying tumor. Imaging at 2 years revealed mild tumor growth with obstruction of the foramen of Monro, enlargement of the cystic component of the tumor, and entrapment of the ipsilateral ventricle. Surgical resection was therefore advised after multidisciplinary team discussion.

**Figure 2:**
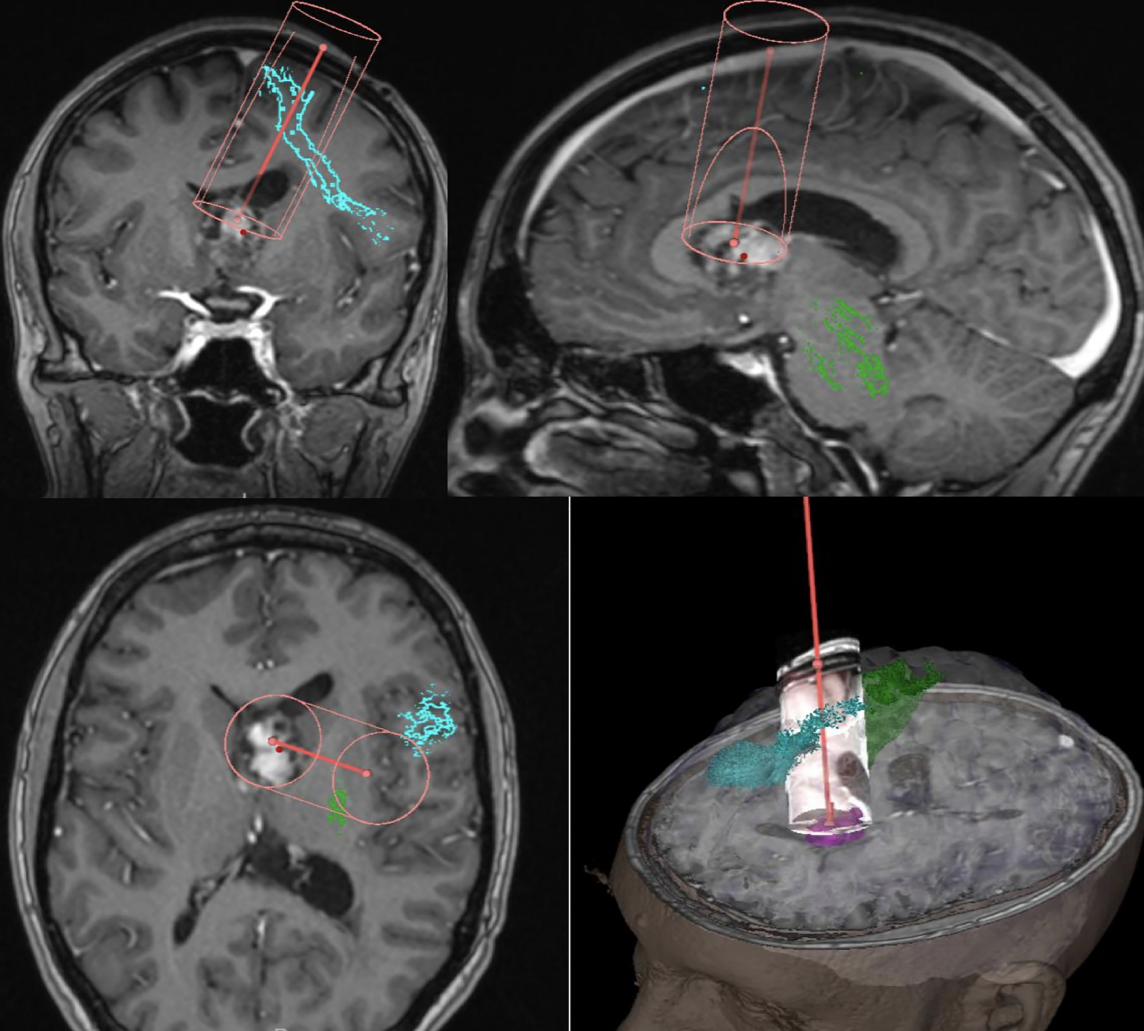
Pre-operative mapping and trajectory planning, Green – CST (cortico spinal tract), Blue – FAT, Magenta – Tumor.

Surgical resection was performed with pre-operative tractography and mapping (Figures 2A,B) A transsulcal minimally invasive parafascicular approach (tsMIPS) was carried out using NICO BrainPath system^©^ with assistance of neuronavigation, 5-ALA, intraoperative neurophysiological monitoring (IONM), and intraoperative ultrasound. Intraoperative transdural mapping of the primary motor cortex confirmed the cortical location of both upper and lower limbs.

**Figure 3:**
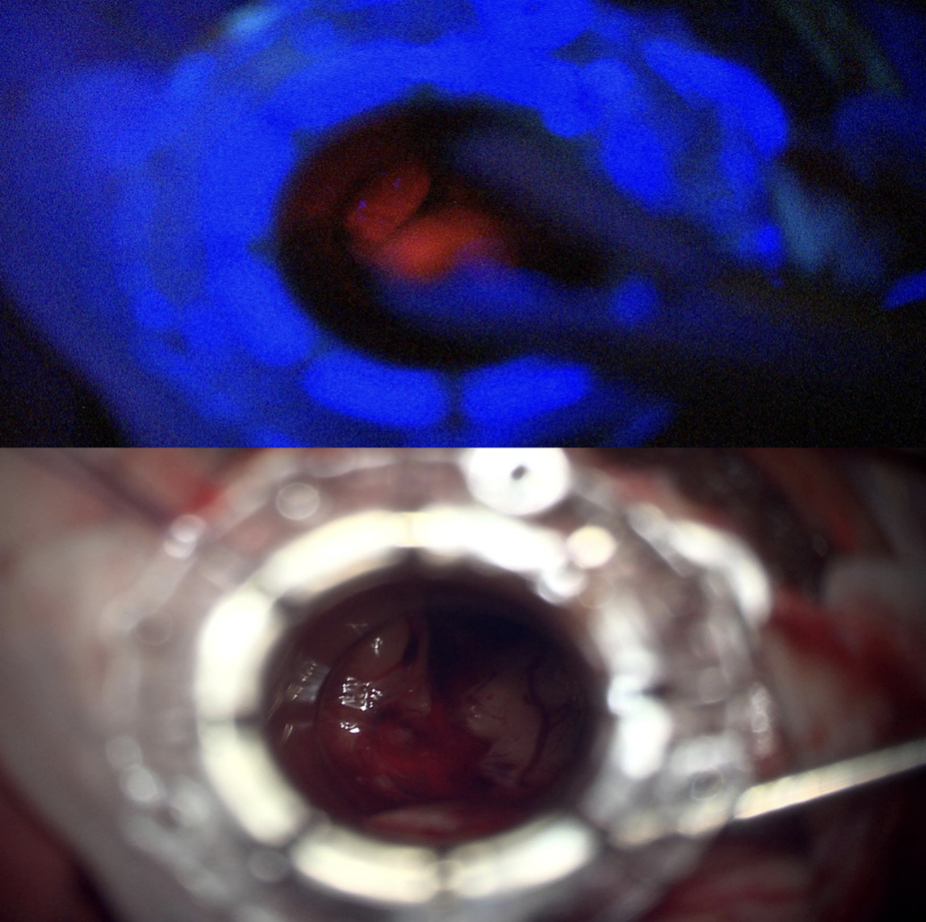
Tumor showing part bright Fluorescence intraoperatively.

**Figure 4:**
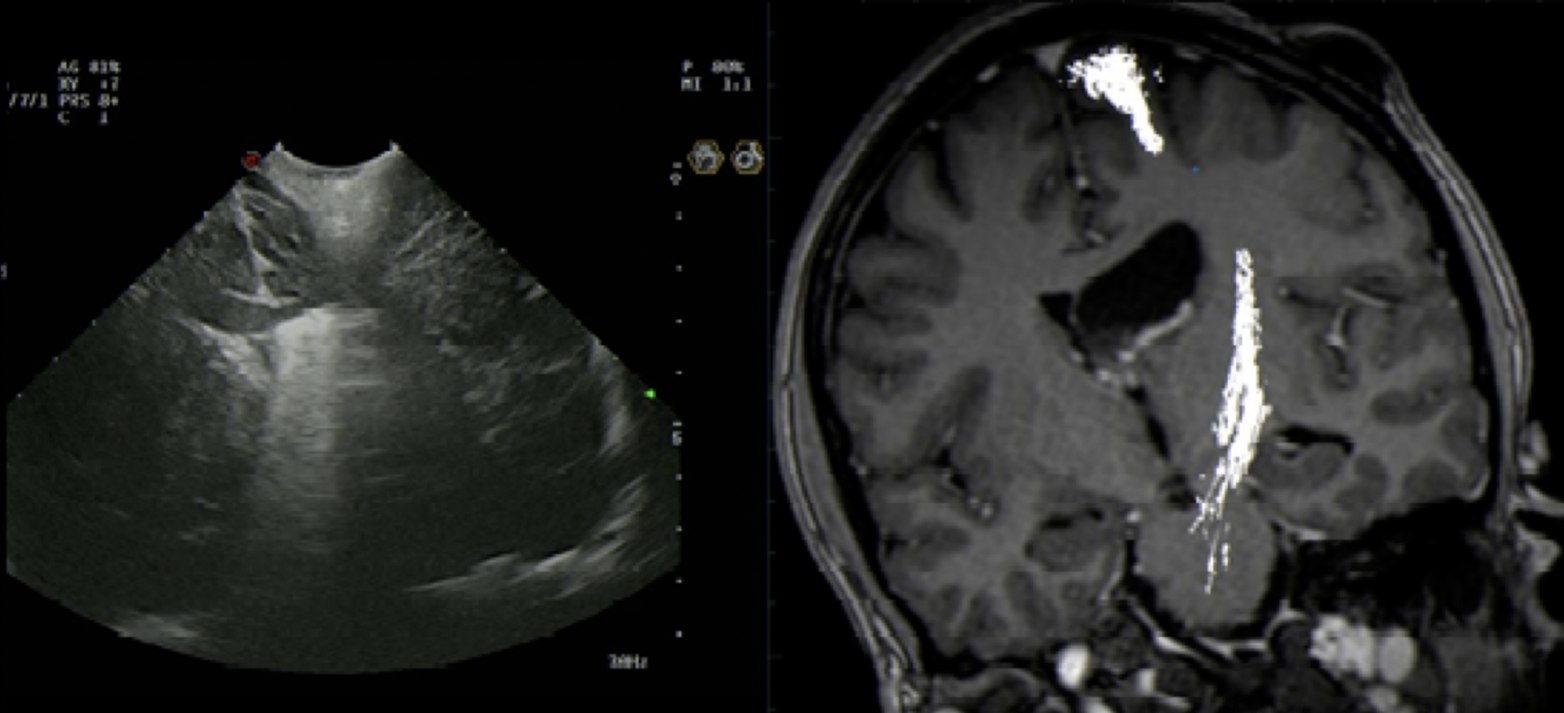
Intraoperative US artefact caused by Residual intraventricular blood and calcified component.

Intraoperatively, the tumor was found to have both bright and pale fluorescence under Blue 400 filter—Zeiss Pentero 900 (Carl Zeiss Meditec®), in the cystic and solid components respectively (Figures 3A,B). A decision was made to limit the resection to the 5-ALA positive tissue and the solid calcified component leaving tumor attached to the walls of fornix, walls of the third ventricle and hypothalamus. No fluorescence was perceived at the end of resection (Figure 4).

**Figure 5:**
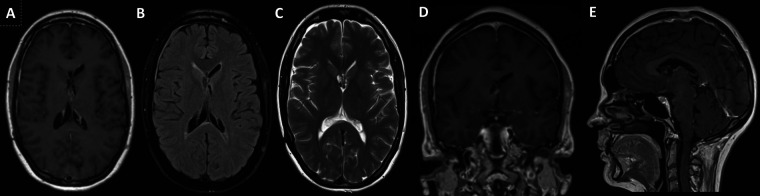
3-Year Follow-up Postoperative MRI – Axial (**A)** – T1-Gad weighted, (**B**) – FLAIR and (**C**) – T2 weighted, Coronal (**D**) - T1-Gad weighted and Sagittal (**E**) - T1-Gad weighted) images showing near total resection with minor contrast enhancing in the lateral walls of the third ventricle and in the fornices. This is the last follow-up scan and the patient has not received further treatment apart from surgery.

The patient made a good recovery with no neurological deficits and post-operative imaging confirmed no complications or evidence of ventriculomegaly and resolution of the entrapped lateral ventricle (Figure 5).

**Figure 6:**
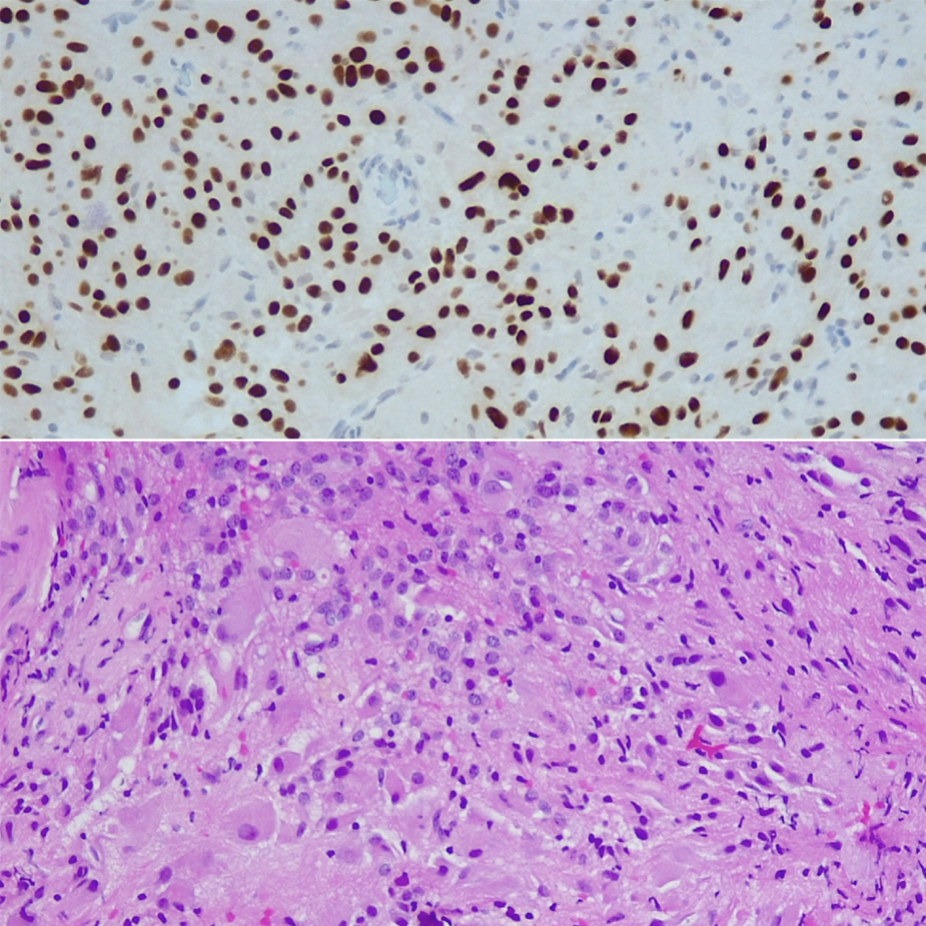
Histology- Areas of epithelioid appearances with large vesicular nuclear and prominent nucleoli associated with abundant granular or ground glass cytoplasm, merged with spindle-shaped cells forming compact glial fibrillary background. Focal calcification visible, tumor also attached to ependymal cells.

Histopathology confirmed Subependymal Giant Cell Astrocytoma (SEGA, WHO grade I). (Figure 6) No adjuvant treatment was required and the patient was under follow-up of neuro-oncology multidisciplinary team with last follow up three years after surgery with stable residual component and no symptoms reported.

## Discussion

SEGA is a rare tumor and appears approximately in 20%–27.4% (4) of patients with tuberous sclerosis. Increase in seizure frequency (15.8%), behavioral disturbance (11.9%), regression/loss of cognitive skills (9.9%) were identified as frequent symptoms associated with SEGA, over and above headaches (8.4%), typically associated with raised intracranial pressure (13).

Extent of tumor resection with no neurological deficits can be technically difficult due to the location and adherence to surrounding tissue and the nature of the tumor, which is usually calcified (14). As with the majority of cases, the tumor in our patient was located in periventricular region, in proximity to the foramen of Monro, and close to subgenual area and the fornix. This made it equally important and challenging for safe resection without damaging important equivocal areas as resection of these lesions can be associated with significant complications particularly in larger lesions – up to two thirds in lesions larger than 3 cm (5). Therefore, a decision towards a fluorescence-guided resection of the higher-grade component was taken to preserve patient's quality of life.

5-ALA was used in our case to target the surgical removal as subtotal resection was planned to preserve the patient's cognition and neurophysiological function. Fluorescence for low grade tumors has been reported with variable results - 8% (15) 9% (16) 16% (the largest published series) (17) 33% (18) and 40% (19) and therefore difficult to generalize. In addition, some benign lesions have been recently reported by our colleagues as fluorescent with 5-ALA, particularly endodermal cysts and glioneuronal tumors (20). Recently, we have successfully presented this case report as a poster at British Neuro-Oncology Society (BNOS) conference in Liverpool (21).

To the best of our knowledge, there have been only two case reports published so far which includes a total of 3 cases mentioning the use of 5-ALA (Gliolan®) in the resection of SEGA (22, 23) (Table 1). Interestingly, two out of three were reported as bright on fluorescence whereas the remaining one was pale. The extent of resection was reported as gross total resection (GTR) for one case whereas there was no data for the rest of two cases. The case with GTR was reported as having no post op complications.

**Table 1:** Operative aspects of cases of SEGA resected with 5-ALA (gliolan^®^).

Publication	Year of publication	Total SEGA cases	SEGA expressing fluorescence	Remarks
Intraoperative Photodynamic Diagnosis of Brain Tumors Using 5-Aminolevulinic Acid. Diagnostic Techniques and Surgical Management of Brain Tumors. IntechOpen, 2011 S. Utsuki, et al.	2011	2	1	– Nil Age/EOR Or post-op neurological deficit data published – Case 1 bright and Case 2 pale fluorescence pattern reported.
Lighting up the Tumor—Fluorescein-Guided Resection of Gangliogliomas J. Höhne, et al.	2020	1	1	– Gross total resection (GTR) – No neurological deficit post op – Bright fluorescence pattern

There was limited data to report any variation in fluorescence brightness within the tumor, which we aim to report here. In our case, we found that the soft or cystic part of the tumor showed higher fluorescence compared to the calcified part. Metabolic imaging was not available for this patient. Further research should focus on the relation between intraoperative 5-ALA fluorescence and preoperative metabolic imaging, such as PET-FGD or PET-amino acids.

The reasons for the low degree of fluorescence of the calcified component are not completely understood. There was a systematic review published in 2016 which evaluated the relationship between the 5-ALA fluorescence and tumor histopathology (24). In a study of Grade II and Grade III Gliomas, it was reported that 5-ALA is a reliable marker for tumor anaplasticity (sensitivity: 89%; specificity: 88%; ppv:85%; npv: 91%) (25). Further there was another study to support this which found that the intensity of fluorescence is representative of the degree of malignancy within the tumor (17). These findings of increased fluorescence rates in high grade gliomas compared to low grade allow us to reasonably conclude that fluorescence positivity is related to higher degree of malignancy. Hence by looking at these studies, we can hypothesize the reason for low uptake of 5-ALA (Gliolan®) in the calcified portion to be the area of low degree of malignancy.

In this case, 5-ALA guided-resection was not used to improve the extent of resection but to target specific areas within the tumor related with potentially higher proliferation areas given our knowledge of 5-ALA metabolism. Despite the use of a minimal invasive approach to resect this tumor, the extent of resection was not limited by this approach. Even if different surgical approaches have been used – such as transcallosal or transcortical, the decision to limit the extent of resection was based on the risk of neurological injury and not on lack of visualization, maneuverability or resectability.

The MIPS approach integrated with the preoperative planning provided by tractography and intraoperative neuromonitoring and mapping allowed for a safer resection and successful preservation of the patient's quality of life.

## Conclusion

5-ALA-guided resection is a potential adjunct not only for maximizing surgical resection but also to target focal subtotal resections in eloquent areas. Its use in SEGA has been rarely reported. Therefore, further studies in the area of photodynamics will be helpful to fully evaluate the efficacy of 5-ALA in SEGA and other low-grade tumors.

## Data Availability

The raw data supporting the conclusions of this article will be made available by the authors, without undue reservation.
